# Metabolic Changes Induced by Purinergic Signaling: Role in Food Intake

**DOI:** 10.3389/fphar.2021.655989

**Published:** 2021-04-29

**Authors:** Vanni Caruso, Mariachiara Zuccarini, Patrizia Di Iorio, Ishaq Muhammad, Maurizio Ronci

**Affiliations:** ^1^ School of Pharmacy and Pharmacology, University of Tasmania, Hobart, TAS, Australia; ^2^ Institute for Research on Pain, ISAL‐Foundation, Rimini, Italy; ^3^ Department of Medical, Oral and Biotechnological Sciences, University of Chieti-Pescara, Chieti, Italy; ^4^ Center for Advanced Studies and Technologies (CAST), University of Chieti-Pescara, Chieti, Italy; ^5^ Department of Pharmacy, University of Chieti-Pescara, Chieti, Italy

**Keywords:** purinergic receptors, food intake, metabolic homeostasis, orexigen and anorexigen neurons, obesity

## Abstract

The purinergic signalling has a well-established role in the regulation of energy homeostasis, but there is growing evidence of its implication in the control of food intake. In this review, we provide an integrative view of the molecular mechanisms leading to changes in feeding behaviour within hypothalamic neurons following purinergic receptor activation. We also highlight the importance of purinergic signalling in metabolic homeostasis and the possibility of targeting its receptors for therapeutic purposes.

## Introduction

The purinergic system consists of a ubiquitous and complex network of intracellular and extracellular components that mediates cell growth and differentiation, neuroprotection, inflammation, and several neuroendocrine functions including energy homeostasis and food intake (Coccurello and Volonté, 2020; Jain and Jacobson, 2020). The regulatory role of the purinergic system is determined by the activity of adenine- and guanine-based compounds, their converting enzymes, as well as P1 and P2 receptors (Burnstock, 2011; Yegutkin, 2014). Specifically, P1 include four adenosine (Ado) receptors (A1, A2A-B, A3), whereas P2 receptors bind both nucleotides and nucleotide sugars (ATP, ADP, UTP, UDP, UDP-glucose) (Fredholm et al., 2011). A_1_ and A_3_ metabotropic receptors couple to the G_i/o_ family and inhibit cyclic AMP (cAMP) production, whereas A_2A_ and A_2B_ are stimulatory G_s_-protein coupled receptors enhancing cAMP production; P2X_1-7_ receptors are ligand-gated ion channels that, following ATP binding, open the pore permeable to Na^+^, K^+^, and Ca^2+^; P2Y_1-2-4-11_ are metabotropic receptors activating phospholipase C (PLC)/inositol triphosphate (IP3)/Ca^2+^ axis *via* G_q_/G_11_ proteins whereas P2Y_12–13–14_ are coupled to G_i_/G_o_ (Burnstock, 2020). After being released in the extracellular milieu, ATP is hydrolyzed to Ado *via* a sequential series of enzymatic reactions catalyzed by several ecto-nucleotidases: ecto-nucleoside triphosphate diphosphorylases (CD39), ecto-5′-nucleotidase (CD73), ecto-nucleotide pyrophosphatase/phosphodiesterases (NPP) and alkaline phosphatases (APs) (Zimmermann et al., 2012; Losenkova et al., 2018). Extracellular nucleosides are, then, taken up by the cells *via* equilibrative nucleoside transporters (ENTs) and concentrative nucleoside transporters (CNTs) and ultimately interconverted to generate purine nucleotides by *de novo* synthesis or *via* the purine salvage pathway.

The dysregulation of the purinergic signaling has been associated with important pathophysiological conditions including neurodegenerative diseases, cancer, inflammation and metabolic disorders such as obesity (Tozzi and Novak, 2017; Burnstock and Gentile, 2018; Boison and Yegutkin, 2019).

P1 and P2 receptors are expressed in metabolically active tissues (e.g., brain, adipose tissue, skeletal muscle, immune system, pancreas, liver) where they regulate gluconeogenesis, inflammation, lipolysis/lipogenesis, insulin sensitivity, energy expenditure, thermogenesis and food intake (Table 1). For an exhaustive review, (Jain and Jacobson, 2020).

**TABLE 1:** Purinergic receptors in food intake and cell metabolism.

Receptor	Endogenous agonists	Functional role	References
A1	Ado	Adipogenesis, lipolysis, lipogenesis, glycogenolysis, gluconeogenesis, energy expenditure, feeding, obesity	González-Benítez et al. (2002), Barakat et al. (2006), Faulhaber-Walter et al. (2011), Gnad et al. (2014), Tozzi and Novak (2017), Wu et al. (2017)
A2A, A2B	Ado	Thermogenesis, adipogenesis, lipolysis, lipogenesis, glycogenolysis, gluconeogenesis browning, insulin homeostasis, hepatic inflammation, regulation of food intake	González-Benítez et al. (2002), Krügel et al. (2003), Yasuda et al. (2003), Carmen and Víctor (2006), Greenberg et al. (2006), Johansson et al. (2007), Gharibi et al. (2012), Kusminski et al. (2016), DeOliveira et al. (2017), Tozzi and Novak (2017), Cai et al. (2018), Gnad et al. (2020), Sacramento et al. (2020)
P2X2	ATP	Metabolic homeostasis (orexigenic effect)	Lee et al. (2005), Wollmann et al. (2005), Florenzano et al. (2006), Colldén et al. (2010), Sun et al. (2012), Li et al. (2015), D'Alimonte et al. (2017), Wang et al. (2020)
P2X5	ATP	Thermogenesis	Nascimento et al. (2014)
P2X7	ATP	Inflammation, adipocyte hypertrophy, dyslypidemia, obesity	(Beaucage et al. (2014), Coccurello and Volonté (2020)
P2Y1	ADP	Regulation of food intake, leptin production, glucose-stimulated insulin response, adipogenesis	Léon et al. (2005), Seidel et al. (2006b), Kittner et al. (2006), Laplante et al. (2010)
P2Y2	ATP; UTP	Release of pro-inflammatory cytokines (MCP-1, CD68, adipocytokines), glucose homeostasis, obesity, adipogenesis, insulin sensitivity	Laplante et al. (2010), Tozzi and Novak (2017), Merz et al. (2018), Zhang et al. (2020)
P2Y4	ATP, UTP	Adipogenesis	Tozzi and Novak (2017)
P2Y6	UDP	Regulation of food intake, glucose uptake, diet-induced obesity, inflammation, insulin resistance	Balasubramanian et al. (2014), Steculorum et al. (2015a), Steculorum et al. (2017b), Jain et al. (2020)

Purinergic receptors are ubiquitously expressed in the central nervous system (CNS) including the hypothalamus, an integral part of the limbic system consisting of a complex architecture of neurons organized in small nuclei that are involved in the regulation of several neuroendocrine functions (Lechan, 2016), including the control of food intake (Timper and Brüning, 2017).

Activation of Agouti-related peptide (AgRP) neurons, a small subset of neurons in the hypothalamic arcuate nucleus (ARC), potently promotes rapid feeding (Aponte et al., 2011), whereas ablation of AgRP neurons results in satiety (Gropp et al., 2005).

The abundant expression of purinergic receptors in the ARC, lateral hypothalamus (LH), paraventricular nucleus (PVN) and, specifically, in hypocretin/orexin neurons, suggests that the purinergic system may play a major role in the regulation of food intake (Florenzano et al., 2006). Anatomically, an abundant expression of P2X_2,4,6_ receptors is found in the neurons of the ARC, whereas a similar receptorial density of P2X_1-6_ receptors is expressed in the PVN where ATP release elicits fast excitatory synaptic transmission (Cham et al., 2006).

Noteworthy, recent studies highlighted the coordinated action of different brain cells (tanycytes, astrocytes, microglia) as well as neuronal-glial interactions in the orchestration of energy homeostasis Andermann, M. L., and Lowell, B. B. (2017). Toward a wiring diagram under-standing of appetite control. Neuron, 95 (4),757–778. https://doi.org/10.1016/j.neuron. 2017.06.014). Astrocytes and microglia are secretory cells that release neuroactive compounds, including purines, in the extracellular milieu, thus contributing to regulate synaptic plasticity and cell adaptation under different stimuli (Peña-Altamira et al., 2018; García-Cáceres et al., 2019). Beyond their well-documented role of structural support and neurotransmission, astrocytes and microglia have been drawing attention for their effect in nutrients and hormone sensing, by virtue of the expression of purinergic, GABAergic and Toll-like receptors (Kettenmann et al., 2011; García-Cáceres et al., 2016). Accordingly, it has been reported that an hypercaloric diet enhance astrogliosis in the ARC, thus suggesting a role of these cells in the pathogenesis of obesity (Balland and Cowley, 2017).

In the present review we illustrate the state-of-the-art of purine modulation of food intake, by taking into account the complex interaction between purinergic signaling with hormones and brain circuits within the hypothalamus and the surrounding regions.

### Role of Purinergic Signalling in Food Intake

Food intake is the result of metabolic, autonomic, environmental and neuroendocrine factors integrated within the hypothalamus, the central hub regulating energy homeostasis (Bernardis and Bellinger, 1996). There is a compelling evidence that purinergic receptors have highly overlapping expression patterns as well as binding profiles in hypothalamic regions (Abbracchio et al., 2009).

Neurophysiologic findings demonstrated that ATP administration on hypothalamic slices induced a dose dependent increase in spike frequency of orexin neurons (Wollmann et al., 2005) and dorsomedial hypothalamic neurons (Matsumoto et al., 2004) and that the entire population of orexigenic neurons express the purinergic subtype receptor P2X_2_R (Florenzano et al., 2006). Specifically, strong P2X_2_R immunoreactivity is found in cell bodies of orexigenic NPY/AGRP/GABA neurons in the ARC (Colldén et al., 2010).

The latest and most specific evidence regarding the potential therapeutic usage of purinergic compounds in obesity arises from physiological studies at the receptor level using transgenic mice and synthetic ligands. Recent evidence of the involvement of the purinergic system in the regulation of food intake suggest that also the UDP-activated P2Y6R is expressed in AgRP neurons (Steculorum et al., 2015b). In obesity, hypothalamic UDP concentrations are elevated as a result of an increased circulating source of uridine, and this elevation might overstimulate feeding *via* P2Y6-dependent activation of AgRP neurons (Steculorum et al., 2015b). The development of selective antagonists for purinergic receptors has corroborated the evidence that pharmacologic inhibition of P2Y6R signaling in AgRP neurons reduces food intake and improves systemic insulin sensitivity in obese mice (Steculorum et al., 2017a).

**FIGURE 1:**
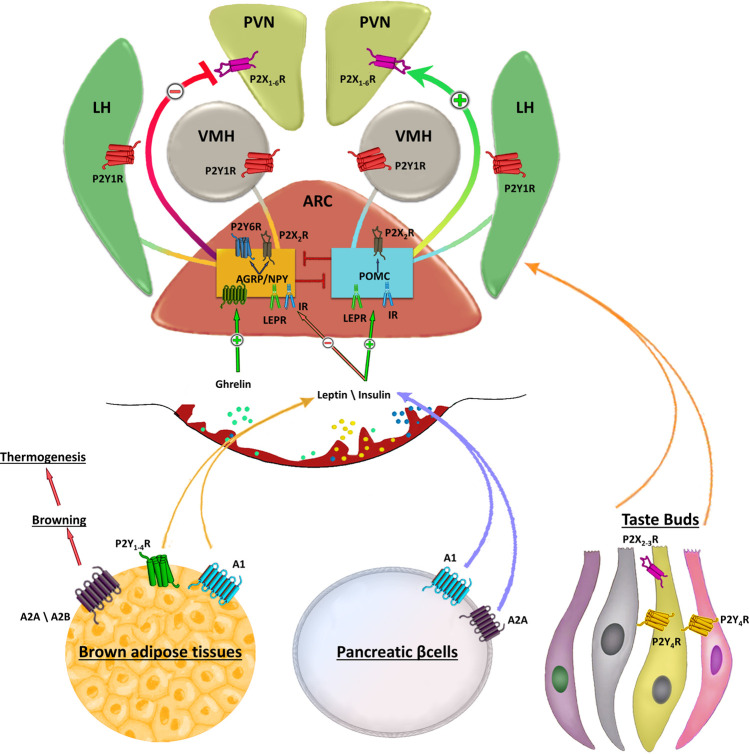
Schematic representation of purinergic signalling in hypothalamus. In the hypothalamus, purinergic signalling is involved in several complex aspects regulating food intake. Endogenous appetite stimulants such as ghrelin promotes food intake inactivating the anorexigenic proopiomelanocortin (POMC) neurons activity, while leptin inhibits the orexogenic signalling of AgRP/NPY neurons. Purinergic receptors are abundantly expressed in the ARC, paraventricular nucleus (PVN), lateral hypothalamus (LH). Strong P2X2R immunoreactivity is found in cell bodies of orexigenic NPY/AGRP/GABA neurons in the ARC and only occasionally in cell bodies of neurons expressing anorexigenic peptides. AgRP neurons also express UDP-activated P2Y6R. The ventromedial (VMH) and lateral hypothalamus (LH) are brain regions with antagonistic functions in the regulation of food intake in which activation of VMH neurons inhibits feeding, whereas stimulation of LH neurons enhances food intake. Peripheral stimulation of purinergic receptors in brown adipose tissue, pancreatic β-cells or taste buds regulates the circulating levels of leptin, insulin and other factors involved in food intake. Stimulation of A2A/A2B receptors induces browning of adipose tissue that in turn increases thermogenesis thus preventing fat accumulation.

Functional studies in animal models have produced exciting discoveries on the role of purinergic signaling in the regulation of food intake. Changes in feeding conditions, from ad libitum to intermittent restriction, have proved to alter the hypothalamic P2Y1 receptor expression in rats (Seidel et al., 2006a). Immunohistochemical staining indicated that P2Y1 receptors and neuronal nitric oxide synthase (nNOS) co-localize in neurons of the ventromedial hypothalamic nucleus (VMH) and LH (Kittner et al., 2006), two functionally antagonistic regions involved in the regulation of food intake (Timper and Brüning, 2017) in which activation of VMH neurons inhibits feeding, whereas stimulation of LH neurons enhances food intake (Brown et al., 1984; Takaki et al., 1992). A direct coupling between purinergic signaling and NOS activity during adaptive feeding processes was tested in rats with microinjections of P2Y1 agonists into both VMH and LH (Kittner et al., 2006). The authors demonstrated that ATP/ADP, acting as extracellular signal molecules in the rat brain, are involved in the regulation of food intake, plausibly depending on P2Y1-receptor-mediated nitric oxide production (Kittner et al., 2004) (Figure 1).

It has been very recently reported that adenosine receptors may play a central role in the management of obesity and metabolic disorders (D'Antongiovanni et al., 2020; de Oliveira et al., 2020). Stimulation of A2A and A2B receptors by specific agonists increased lipolysis and brown adipose tissue (BAT) thermogenesis, and protected mice from diet-induced obesity (Gnad et al., 2014). Increasing thermogenesis *via* the metabolic activity of BAT has been considered as a pharmacological intervention able to fight the energy imbalance underlying weight gain and obesity (Gnad et al., 2014). Therefore, the thermogenic/lipolytic effects of Ado *via* activation of BAT and, subsequently, fat catabolism, could be a promising approach to address metabolic disorders. An increased expression of A1R in thermoregulatory neurons has been associated with obesity in mice, whereas stimulatory doses of the purine alkaloid caffeine, a non-selective A1R antagonist, was able to decrease body weight and increase brown adipose tissue (BAT) thermogenesis in rats fed a HFD (Collden et al., 2010; Wu et al., 2017). Research efforts have also provided a direct evidence that adenosine receptors in hypothalamic glia cells could play a role in feeding responses (Yang et al., 2015). Combined chemical genetics, cell-type-specific electrophysiology, pharmacology, and feeding assays demonstrated that stimulation of astrocytes within the medial basal hypothalamus reduces both basal- and ghrelin-evoked food intake (Yang et al., 2015). Specifically, activation of A1 receptors mediated the astrocytic inhibition of food intake and the firing rate of AGRP neurons suggesting that the glial circuit could be a novel target for therapeutic intervention in the treatment of appetite disorders (Yang et al., 2015).

Recent interest on the role of glia cells in food intake focuses on the roles of hypothalamic tanycytes, chemosensitive glial cells with a unique morphology. Hypothalamic tanycytes are in contact simultaneously with the cerebrospinal fluid (CSF) in the third ventricle and with major neural populations in the hypothalamic parenchyma (Bolborea and Dale, 2013; Goodman and Hajihosseini, 2015). Physiologically, tanycytes can sense nutrients such as glucose and amino acids in the CSF evoking robust ATP-mediated Ca2+ responses (Frayling et al., 2011; Orellana et al., 2012). The release of ATP in response of glucose or amino acids results in the activation of purinergic receptors in hypothalamic neurons of the arcuate and ventromedial nucleus (Bolborea et al., 2020). Specifically, optogenetic studies demonstrated that tanycytes can activate purinergic receptors in orexigenic NPY-expressing neurons in the ARC to induce acute hyperphagia when activated by light (Bolborea et al., 2020). Taken together, tanycytes sense the elevation of glucose and amino acids in plasma and CSF following a meal, and in response, they release ATP into hypothalamic neurons activating anorexigenic pathways to reduce appetite.

There is a consensus that ATP and adenosine are also involved in the rewarding effects of feeding in a functionally antagonistic manner (Krügel et al., 2003; Kittner et al., 2004).

Animal studies demonstrated that stimulation of ADP/ATP sensitive P2 receptors in the nucleus accumbens (NAc), a primary site mediating reward behaviour, reinforced their dopaminergic responses and enhanced food intake (Krügel et al., 2001), while the blockade of P2 receptors decreased their feeding responses associated with dopamine release (Kittner et al., 2000). In an elegant behavioural study where microdialysis was combined with encephalographic measurements, injections of non-selective P2 and P1 receptor antagonists in the NAc of rats, PPADS and 8-SPT respectively, interacted antagonistically in the regulation of feeding behaviour and feeding-induced changes of EEG activity (Kittner et al., 2004). Other evidence indicate that adenosine suppressed dopamine release *via* agonism of the A_2A_ receptors in the NAc and this was accompanied with the reduction in food intake (Krügel et al., 2003), whereas the agonism of the A1 receptor was not involved in feeding responses (Krügel et al., 2003; Mingote et al., 2008). This might suggest that selective blockage of purinergic receptors in the NAc modulates the rewarding effects of feeding behaviour. Beyond the established hypothalamic-mesolimbic pathway circuitry for the regulation of food intake, a diverse array of detectors in the oral cavity including taste receptors in the tongue play a pivotal role in the modulation of energy homeostasis mechanisms (Chaudhari and Roper, 2010; Depoortere, 2014).

Taste buds are a collection of gustatory sensory cells that release ATP, among other neurotransmitters such as acetylcholine, serotonin, norepinephrine or GABA in response to gustatory stimulation (Khan et al., 2020). The release of these molecules enhance the communication with the gustatory centre of the brain (i.e. the insular cortex and then hypothalamus) through cranial nerves including the glossopharyngeal nerve, the facial nerve and the vagus nerve (Frank and Hettinger, 2005). Specifically, in response to gustatory stimulation, ATP and neurotransmitters are released to enable chemical signalling within the taste bud itself or with afferent sensory nerves that express P2X2/P2X3 receptors on the nerve fibers innervating the taste buds. (Chaudhari and Roper, 2010).

Taste buds are divided in four morphological subtypes: Types I, II, III, and IV and among these subtypes, type II cells are the most characterised (Nelson et al., 2001; Depoortere, 2014). ATP is released by Type 2 cells in response to sweet, bitter or umami testants (Besnard et al., 2016) and genetic inactivation of P2X2/P2X3 receptors in nerve fibres is associated with decreased salty and sour tastes (Finger et al., 2005). Once released, ATP can also activate adjacent Type 3 cells triggering the release of serotonin which contribute to prolong the transmission of the taste signals to the brain (Besnard et al., 2016).

In the obese, the number and density of the taste buds is reduced by 25% compared to healthy individuals suggesting that overeating could be associated with impairments in purinergic afferent reward-induced signalling (Proserpio et al., 2015; Coccurello and Maccarrone, 2018; Kaufman et al., 2018).

## Discussion

During the past 4 decades, purinergic signalling has received considerable attention regarding its involvement in the fine regulation of food intake. The advent of new molecular tools, conditional knockout strategies targeting specific neuronal populations as well as animal behavioural models have shed further light on this function. For example, since when hypothalamic gliosis was associated with inflammation resulting from high-fat diet feeding in both rodents and human (Thaler et al., 2012), several investigations on non-neuronal cells have since been reported in energy homeostasis and obesity pathogenesis (Douglass et al., 2017). There are direct evidence that adenosine receptors, in particular A1R, in hypothalamic glia cells play a role in feeding responses (Yang et al., 2015), as endogenous Ado inhibited basal food intake and counter-regulated the ghrelin-elicited feeding by inactivating the orexigenic AGRP neurons in the ARC.

Moreover, nutrient sensing tanycytes activate the arcuate neuronal network releasing ATP and promoting acute hyperphagia (Bolborea et al., 2020). It has been demonstrated that the long-term exposure to high fat diet induces hypothalamic gliosis (Douglass et al., 2017) and given the dramatic increase in childhood obesity, the question whether homeostasis-challenging circumstances on purinergic signalling early in life could predispose to a multifactorial and complex disease in adulthood, is still a matter of debate.

Purinergic signalling also plays a major role in the regulation of peripheral sensory pathways of the gustatory system for the regulation of food intake (Besnard et al., 2016). To date, the majority of anti-obesity agents targeting signalling pathways in metabolic tissues such as liver, adipocytes and skeletal muscles have failed to deliver significant clinical results (Rodgers et al., 2012). Targeting the gustatory signalling pathways could represent a promising and effective strategy that can provide clinically relevant anti-obesity agents.

The protection from diet-induced obesity through the thermogenic/lipolytic effects of Ado, may be mediated by the metabolic activity of BAT *via* the autonomic nervous system stimuli originating from the dorsomedial hypothalamic nucleus (DMN) in a loop mechanism.

The multiple roles of the purinergic signalling in the regulation of food intake are both an opportunity for therapeutic interventions, but also a concern when considering the risk of side effects of a new compound. Noteworthy, the translation from studies in mice to clinical trials in humans is still a big challenge due to many factors, including the heterogeneity of the cells forming the neuronal circuits which are difficult to study singularly and attribute them an univocal function separated from the dynamic microenvironment, as well as the ubiquitous expression of purinergic receptors that, in the absence of specific agonist/antagonist, generate compensatory mechanisms blurring their specific role. The neuro-anatomical interactions of purinergic signalling within hypothalamic circuits and the nucleus accumbens might suggest the design of multifunctional compounds able to target their respective receptors separately, which may result in a greater therapeutic effect for the cure of obesity and immunometabolic disorders. Among others, P2Y6R, P2X7R or A1 specific inhibitors may represent novel therapeutic tools in the management of diet-induced obesity.

Taken together, purinergic signalling between brain regions involved in motivation, reward and energy homeostasis present a novel and valid target for the control of feeding behaviour, where selective pharmacological intervention might produce promising results.
